# Might Topical Heparin Help With Occlusion Emergencies After Accidental Intra-Arterial Hyaluronic Acid Injections?

**DOI:** 10.1093/asjof/ojae126

**Published:** 2024-12-14

**Authors:** Marco Stabile, Maurizio Cavallini, Mauro Raichi

## Abstract

Hyaluronic acid fillers rarely cause potentially devastating occlusive adverse events that require immediate hyaluronidase salvage infiltrations. An exploratory photographic investigation probed whether topical heparin's anticlotting and anti-inflammatory properties could synergize with and enhance the effectiveness of hyaluronidase. Based on heparin pharmacodynamics, the authors explored the rationale for associating topical heparins with hyaluronidase in treating occlusive side effects following accidental intra-arterial hyaluronic acid injections. In the first case, an occlusion in the right superior labial artery area, highlighted by reddish-blue net-like skin discoloration (livedo reticularis), developed below the nasal pyramid shortly after 3 intradermal injections of low-viscosity hyaluronic acid gel, rapidly progressing to the glabellar and forehead regions. Within 1 h after the hyaluronidase salvage injection (80 IU), topical low-molecular-weight heparin (40 mg enoxaparin) was uniformly applied, and the procedure was repeated every 8 h for 15 days. In the second case, a cluster of severe occlusive lesions developed in the nose and nasal tip areas after 3 hyaluronic acid injections (formulation and doses as previously stated). After the first week, enoxaparin (4000 IU) was applied topically every 8 h for an additional 3 weeks. Two sequences of photographs document the occlusions’ evolution toward almost complete skin repair after 28 days (first case: immediate combined treatment) and 15 days (second case: sequential treatment spaced 1 week).

The anti-inflammatory and antithrombotic pharmacodynamics of heparin and heparin derivatives offer a promising rationale as an add-on option (combined hyaluronidase and topical heparin) to treat the occlusive side effects caused by hyaluronic acid.

**Level of Evidence: 5 (Therapeutic):**

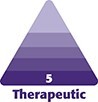

Filler-induced vascular occlusions have a long history. Common evokers of accidental intra-arterial injection include a sudden and frightening blindness or, more frequently, a painful reddish-blue skin discoloration that appears with a dusky, mottled, net-like pattern (livedo reticularis) associated with a slow capillary refill.^1,2^ Immediate reactive hyperemia may occur instead of livedo if the injected material is insufficient to occlude the artery—typically <0.1 mL for the angular artery. Persistent deep blue-black discoloration, blister or bullae development, tissue sloughing, and possibly wounds or abscesses follow these early skin lesions over the next few minutes, hours, and days. The lesions, which may progress to scarring with pigment changes and epidermal and dermal necrosis, are associated with turbulent arterial flow and evidence of obstruction observed in Doppler ultrasound imaging.^1,2^

The earliest reports of unexpected blindness, strokes, and pulmonary emboli date back more than a century. At the beginning of the 20th century, the first reports followed the introduction, in the late 1890s, of poorly biocompatible mixtures based on paraffin and Vaseline injected through large-bore needles. The purpose was to correct and sculpt nasal dorsal augmentations and other facial deformities (Figure 1).^1,3,4^

**Figure 1.**
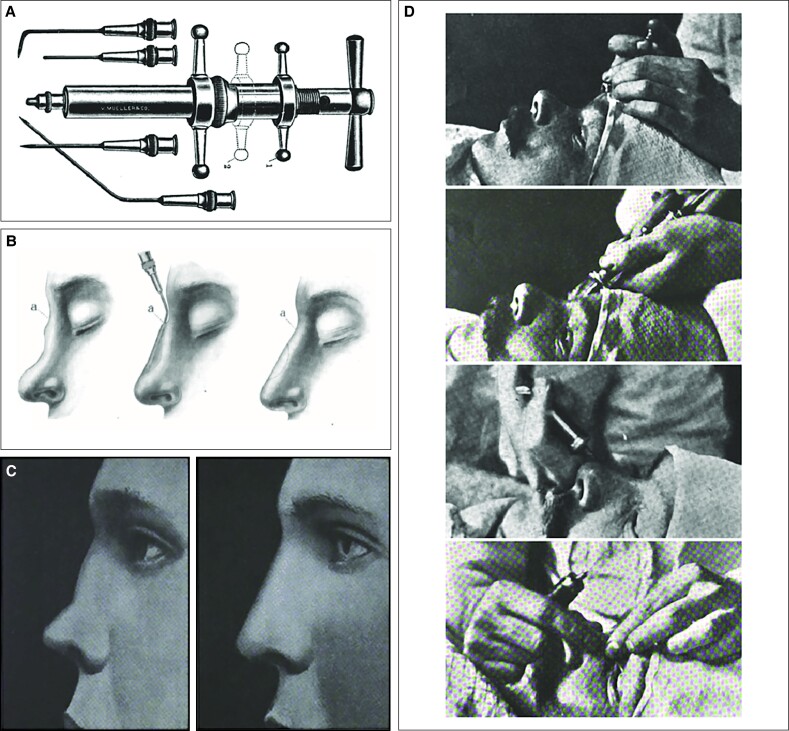
(A) Paraffin syringe used in paraffin facial injections. (B) Diagrammatic description of nasal dorsal augmentation with paraffin, ca. 1911. (C) A patient (age unknown) before and after paraffin augmentation of the nasal dorsum, early ca. 1911. (D) Procedural technique of paraffin injection for correcting a dorsal nasal deformity, ca. 1908. This figure originally appeared within Soares et al, published by MDPI under a Creative Commons Attribution 4.0 International License agreement, which permits reproduction of the image with proper attribution to the original work.^4^

Disfiguring paraffin granulomas disappeared with the dismissal of such techniques in the 1920s. Still, with a low but non-negligible incidence estimated to range between 1 in 2000 and 1 in 10,000, or 0.5‰ to 0.1‰ (somewhat more, 1:6600 or 0.15‰, in a recent Dutch study that accounts for underreporting and underestimation of performed filler treatments), aesthetic medicine specialists are likely to encounter a filler-induced adverse event at least once during their lifelong professional activities.^1^ Overall, the incidence of filler-induced ischemic skin injuries escalated 30-fold between 2000 and 2020, whereas the incidence of filler-induced strokes rose by 300%, with hyaluronic acid fillers comprising nearly four-fifths of filler products. According to the US-FDA Medical Device Reports, cosmetic filler-associated vascular events between 2015 and 2020 occurred mainly in the perioral and lip area (38%), followed by the nasolabial fold (18%) and nasal areas (10%), and least frequently in the cheek area (8%).^4^

Prompt recognition of the occlusion is crucial, keeping in mind that vasoinoculation may occur directly intraluminally; however, it can also happen indirectly through transarterial penetration (Figure 2).^4^ The treatment mainstay is the immediate, diffuse, subcutaneous infiltration of hyaluronidase in the ischemic area, along with topical nitroglycerin under occlusion, antiplatelet acetylsalicylic acid, massage, and warm compresses.^5-7^ Second-line therapies include intra-arterial hyaluronidase, hyperbaric oxygen therapy, and vasodilating prostaglandin E_1_.^2^

**Figure 2.**
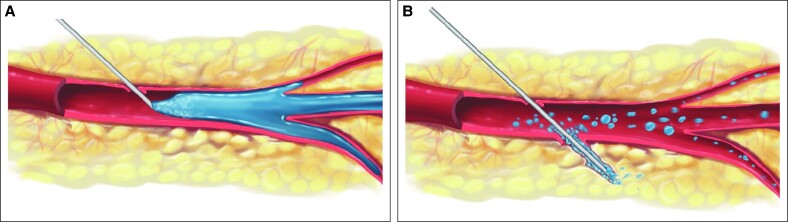
Vasoinoculation by (A) direct intraluminal injection and (B) indirect extraluminal inoculation following transarterial perforation. This figure originally appeared within Soares et al, published by MDPI under a Creative Commons Attribution 4.0 International License agreement, which permits reproduction of the image with proper attribution to the original work.^4^

There may be reasons for the low potential for microvascular occlusion inherent to the pharmacodynamics of hyaluronic acid derivatives. Their heparin-like activity is not a novelty: whole-blood clotting inhibition, Factor Xa and thrombin inactivation related to the degree of sulfation, and the inhibition of von Willebrand factor–dependent platelet agglutination and ADP-induced platelet aggregation.^8,9^ At the same time, research is shedding light on the molecular mechanism of heparin's anti-inflammatory activity, and much evidence supports the extensive cross-talk between hemostasis and inflammation.^10,11^ Conversely, other filler ingredients, different from the available hyaluronic acid derivatives, such as collagen and fat, have clot-promoting activity and can cause more severe occlusions than those caused by hyaluronic acid.^12,13^ The evidence from inflammatory embolia cutis medicamentosa, or Freudenthal–Nicolau syndrome—first described in 1924 as a full-thickness dermal necrosis following the intramuscular injection of an oily bismuth suspension to treat syphilis—shows that any anti-inflammatory activity would be beneficial.^14^

All these considerations were the foundation for the rationale behind the reported exploratory case reports. Could promoting an anticlotting and anti-inflammatory local environment through topical heparin at the site of the developing arterial occlusion synergize and enhance the effectiveness of hyaluronidase? This hypothesis would be innovative, and the combined treatment would be simple, rapid, and cost-effective in emergency salvage situations. Does it have a solid foundation? The photographic case reports presented here explore the idea. Substantiating the hypothesis in a formal, controlled clinical trial would be difficult because of the low incidence of occlusive adverse events associated with hyaluronic acid fillers. Expanding the retrospective case series of patients treated with the hyaluronic acid/topical heparin combination and conducting collateral ex vivo pharmacodynamic investigations will hopefully shed more light on the combination's clinical efficacy and value.

## METHODS

### Occlusive Sequences of Events and Salvage Treatments

#### First Case

A female in her mid-forties developed pain in the right nasolabial fold area, medially, 0.5 to 1 cm below the nasal pyramid, 3 to 4 min after a series of three 0.1 mL intradermal injections of a low-elasticity, low-viscosity biphasic hyaluronic acid gel (20 mg/mL, 4% 1,4-Butanediol diglycidyl ether (BDDE) cross-linking, 30 G needle) in the nasolabial fold area without anesthetic agents. After about 5 min, the painful facial artery territory, which initially seemed slightly paler than the nonpainful surrounding skin at the operator, developed an evident blue-mottled macular discoloration with no evidence of vision loss or oculomotor nerve palsy. At least initially, the normal skin appearance and lack of pain around the alar crease and the nasal alar region seemed to exclude an occlusive involvement of the angular artery cranially to the origin of the superior labial artery. The operator interpreted the sequence of events as secondary to an occlusion limited to the distribution area of the superior labial artery. He also interpreted the arterial occlusion as likely complete in the affected segmental branch because of a lack of reactive hyperemia after the initial skin blanching rather than because of the developing livedo. However, in just a few minutes, the development of glabellar and forehead lesions signaled an upward progression toward the supratrochlear arteries. A prolonged local capillary refill time, with the return to normal pink, warm skin in about 5 s compared with the standard 1 or 2 s, reinforced the clinical suspicion of arterial insufficiency because of filler embolism. An *impromptu* Doppler ultrasonography in the nasolabial area, performed while preparing the salvage hyaluronidase injection, confirmed a severe blood flow deficit.

The female immediately received 80 IU of purified hyaluronidase without dilution with local anesthetics, followed by massage. The hyaluronidase injection technique was standard, perpendicular to the skin, using a 30 G, 10 mm needle; there were no allergic complications.^14,15^ All regulatory and legal prescriptions for the (off-label in Italy) administration of hyaluronidase after a hyaluronic acid injection, including the female's informed consent, were scrupulously followed.^7^

Within an hour after the hyaluronidase salvage injection, the female received 40 mg or 4000 IU, of enoxaparin, a low-molecular-weight heparin, in 0.4 mL topically from a commercial formulation, uniformly distributed using a syringe without a needle over the painful dry area. A delicate massage and rest followed, allowing the area to dry.

The procedure was repeated every 8 h over the 15 day topical treatment period, avoiding contact with water and antiseptic formulations. The authors do not consider any overlay dressing of the topically treated area mandatory; therefore, they left the treated area to air dry, further benefiting from real-time visual monitoring of the healing progress. No antibiotics were administered, nor were nitroglycerin creams applied topically.

#### Second Case

A female in her early thirties, who had undergone elective rhinoplasty 2 years before the hyaluronic acid session, developed severe pain and occlusive lesions in the distribution territories of the angular, columellar, and dorsal nasal arteries (nose and nasal tip) after three 0.1 mL intradermal injections of biphasic hyaluronic acid gel similar to that previously described. She immediately received 100 IU of hyaluronidase. Because of the unsatisfactory evolution of occlusive lesions over the first posttreatment week, the female received 4000 IU of enoxaparin every 8 h starting in the second week for 3 additional weeks, following the same procedures described in the first case report. A sequence of photographs illustrates the evolution of the occlusive skin lesions for both females over their respective follow-up periods.

All performed activities were within the accepted regulatory indications for ambulatory hyaluronic acid treatments, as stated in the Patient Information Leaflet. The hyaluronidase treatment for occlusive skin adverse effects is officially regarded as off-label in Italy. Still, it is legal to perform the procedure in the office under the operator's responsibility. These considerations allow for waiving formal preliminary approval by IRB.

## RESULTS

### First Case

**Figure 3.**
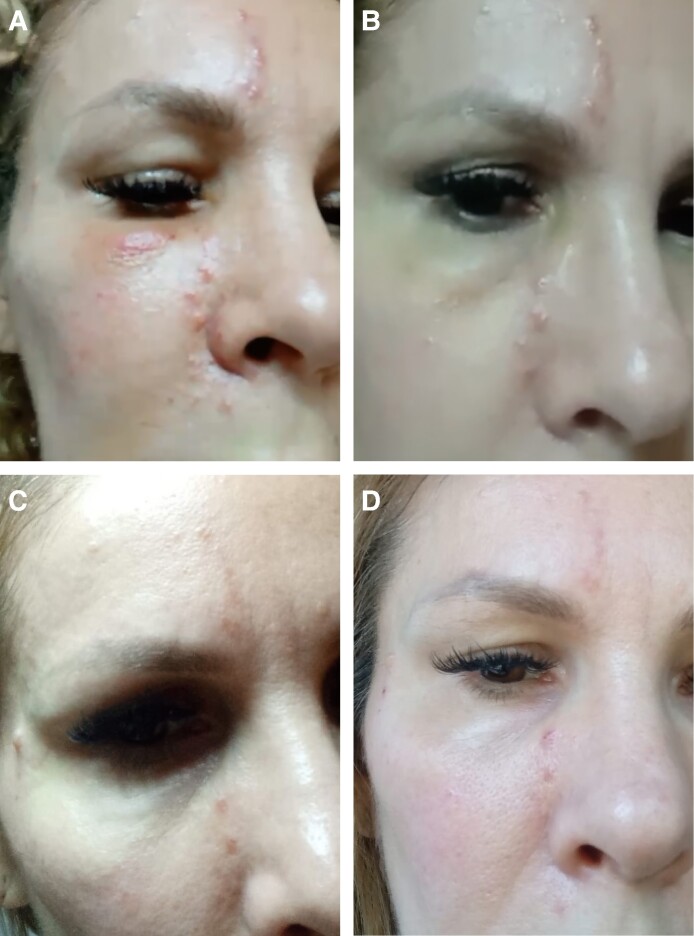
Occlusive lesions in a 43-year-old female at baseline, immediately before starting the combined hyaluronidase and topical heparin treatment (A); favorable evolution after 3 and 7 days (B and C, respectively), and highly satisfactory aesthetic outcomes after 1 more week of combined therapy (D).

Figure 3 documents the baseline presentation of the occlusive skin lesions 2 h after the same-session topical low-molecular-weight heparin application (Figure 3A), the evolution over the first week (Figure 3B, C), and the almost complete skin repair after 15 days (Figure 3D). The pain and edema disappeared after the first and third topical applications.

### Second Case

**Figure 4.**
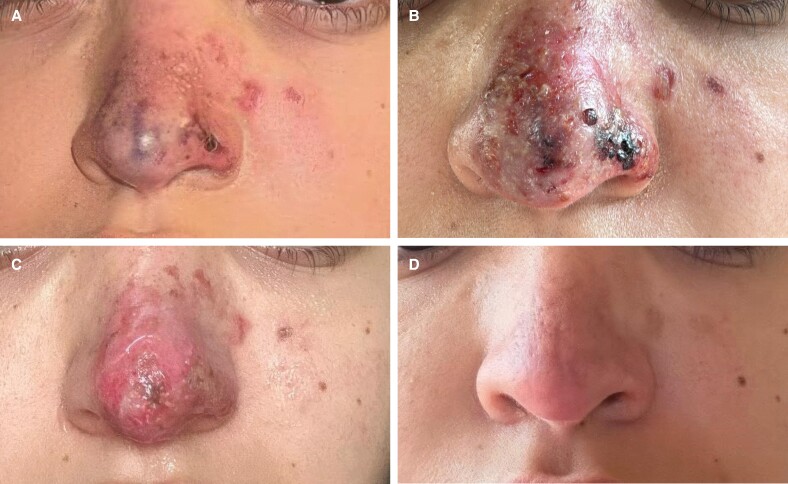
(A) Baseline presentation of occlusive lesions in a 34-year-old female a few minutes after the hyaluronidase injection; (B) a worsening situation after 3 days and slow, unsatisfactory improvement after 7 days (C), vs highly satisfactory aesthetic outcomes on the 28th day (D) after starting topical enoxaparin (early second week) and applying it 3 times daily for 3 additional weeks.

Figure 4 illustrates the severe baseline presentation of the occlusive skin lesions shortly after the hyaluronidase injection (Figure 4A) and the unsatisfactory clinical progression over the first week, with impressive worsening observed on the third day (Figure 4B, C). Figure 4D documents the final, nearly complete skin repair after 3 more weeks of 3-times-daily topical application of low-molecular-weight heparin and a total of 28 days of follow-up.

## DISCUSSION

A sound knowledge of facial vascular anatomy is crucial for treating filler-induced occlusive disorders.^2,5,6^ The topographic anatomy of obstructive lesions depends on the artery involved—the superficial temporal and supratrochlear arteries, respectively, for the temple and forehead; the zygomatic-orbital artery for the lateral eye corner; the angular, columellar, and dorsal nasal arteries for the nose and nasal tip; the facial artery for the nasolabial fold region; the transverse facial and infraorbital arteries, respectively, for the cheek and midface; the superior labial and possibly the columellar arteries for the upper lip; the inferior labial artery for the chin and lower lip; and the submental artery for the chin and tongue.

The adverse events from compromised local arterial distribution—a combination of compression and embolization of the subdermal plexus—mainly occur in 2 areas: the nasal dorsum and nasolabial crease, which depend on the angular and nasal dorsal arteries, and the forehead, eyebrows, and glabella, which rely on the supratrochlear and supraorbital arteries.^2,5,6^ Paradoxically, the extensive collateral pathways—for instance, the nasal anastomoses between the external and internal carotid arteries—can cause tissue embolization both proximally and distally, as well as contralaterally to the injection site.^2^

Even the most recent guidelines have concentrated on the acute salvage treatment of hyaluronidase occlusions, but they do not extend beyond generic suggestions for managing occlusive skin lesions, with limited evidence at best regarding the benefits.^15,16^ The latter is the case for hyperbaric oxygen therapy (HBOT);^2^ conversely, the pathophysiology and molecular dynamics of hyaluronic acid filler-induced occlusive side effects, as well as the allergic risks of hyaluronidase, are receiving closer attention.^3,15^ Preventing skin necrosis may benefit from applying vasodilating warm gauze and 2% nitroglycerin paste, along with administering corticosteroids to reduce swelling and inflammation, acetylsalicylic acid as a platelet antiaggregant, sildenafil and selective inhibitors of cyclic guanosine monophosphate, and systemic heparins to increase local blood flow. Cephalosporins can prevent impetigo, and HBOT can optimize in-depth local oxygenation.^5,17^ Still, the benefits of the proposed addition of other collateral treatments to hyaluronidase, while speculatively rational and substantiated in other indications, are hardly supported by rigorous evidence for inadvertent occlusive disorders secondary to hyaluronic acid injections. The suggested interventions are the same as those proposed for prevention.^5,17^

The pathophysiology of occlusive adverse events with hyaluronic acid dermal fillers is purely mechanical and differs from superficially similar inflammatory occlusive events seen in the past with embolia cutis medicamentosa and those currently observed as a glatiramer complication in the treatment of relapsing-remitting multiple sclerosis.^18^ The arterial occlusions caused by hyaluronic acid dermal fillers differ from those resulting from intravascular clotting, with edematous inflammatory injuries to the arterial lining extending beyond a purely mechanical effect. The pathophysiology of occlusion observed with hyaluronic acid fillers also differs from the arterial occlusions that may occur after accidental injections with collagen- or fat-based fillers, whose primordial activator is the clotting cascade.^12,13^

The degradation rate of hyaluronic acid derivatives in fillers is unlikely to be critical. Degradation rates are variable in vitro following exposure to hyaluronidase and depend on the ingredient concentration and physicochemical properties, namely cross-linking.^19^ Still, such variable properties do not significantly influence the in vivo enzymatic degradation rates beyond the early time points.^20^

Regarding the rationale for the proposed combined hyaluronidase/topical heparin strategy, the anti-inflammatory and antithrombotic pharmacodynamics of heparin and its derivatives provide a sound and supportive rationale.^7-10^ More importantly, from a clinical perspective, there is abundant clinical evidence of the benefits of topical heparin and heparin derivatives in treating severely inflammatory skin conditions, such as burns^21-23^ and skin necrosis associated with septic shock hypoperfusion because of intravascular disseminated coagulation, even as alternatives to surgical debridement.^24^ Speculatively, all conditions secondary to intra-arterial occlusion should benefit from the peculiar topical heparin pharmacodynamics and ease of application as salvage add-on co-treatment, ranging from early pain, skin hyperemia, and livedo reticularis to late necrosis.^10^

Although the rationale for combining hyaluronidase and topical heparin may be sound, the preliminary report of 2 episodic pieces of evidence needs a more rigorous demonstration. Still, the case history of the younger female, with very severe nose lesions up to partial necrosis of the nose tip and showing no tendency to regress after hyaluronidase treatment, is striking and impressive, with almost complete skin repair without scarring in 3 weeks of thrice-daily topical heparin therapy. Moreover, the delayed start of topical heparin therapy in that young female suggests that immediate co-treatment need not be mandatory. Collecting a controlled retrospective case series of combined hyaluronidase/topical heparin vs standard hyaluronidase therapies, as currently initiated by the authors, will further support this innovative, easy-to-perform, and inexpensive add-on strategy for managing and possibly preventing cutaneous occlusive lesions secondary to hyaluronidase injections.

## CONCLUSIONS

Although only a suggestion for the moment, with no incontrovertible clinical demonstration, the anti-inflammatory and antithrombotic pharmacodynamics of heparin and heparin derivatives likely provide a sound rationale for the combined hyaluronidase/topical heparin treatment of occlusive skin side effects because of hyaluronic acid.
